# Exploratory analysis of health-related quality of life among the empty-nest elderly in rural China: An empirical study in three economically developed cities in eastern China

**DOI:** 10.1186/1477-7525-12-59

**Published:** 2014-04-25

**Authors:** Ying Liang, Wei Wu

**Affiliations:** 1 Department of Social Work and Social Policy, School of Social and Behavioral Sciences, Nanjing University, Nanjing 210023, Jiangsu province, People’s Republic of China; 2 School of Social and Behavioral Sciences, Nanjing University, Nanjing 210023, Jiangsu province, People’s Republic of China

**Keywords:** Rural China, Empty-nest elderly, HRQOL, EQ-5D, SF-12

## Abstract

**Background:**

Along with rapid economic development, the aging process in China is gradually accelerating. The living conditions of empty-nest rural elderly are worrisome. As a more vulnerable group, empty-nest elderly are facing more urgent health problems. This study explores the health-related quality of life (HRQOL) of empty-nest elderly in rural China and aims to arouse more social concern for their HRQOL.

**Methods:**

Research subjects were empty-nest rural elderly from three cities: Nanjing, Suzhou, and Wenzhou (ages ≥ 60, *n* = 967). This study used the five-dimensional European quality of health scale (EQ-5D) and the 12-item Short Form Health Survey (SF-12) to measure the HRQOL of the respondents. Spearman correlation coefficient, stereotype logistic regression, ordered probit regression and multinomial logistic regression, and Structural equation model (SEM) methods are employed to study the relationship.

**Results:**

(1) The Spearman correlation coefficient shows that the correlations of similar domains between the SF-12 and the EQ-5D scales are relatively strong. (2) Men’s scores are higher than that of women’s in general health (GH) and anxiety/depression (AD) models. (3) The scores of physical component summary (PCS), physical functioning (PF), mental health (MH), and usual activities (UA) decline with age. (4) Apart from PCS, vitality (VT), and role-emotional (RE) as dependent variables, the education passes all the significance tests. The higher the education is, the higher the scores of physical or psychological health are. (5) The scores of PCS and bodily pain (BP) of empty-nest elderly are divorced or higher in other marital status. (6) In SEM analysis, the effect of basic information of empty-nest elderly on SF-12 scale is more significant.

**Conclusions:**

***First,*** the frequency histograms of EQ-5D show that the scores of empty-nest elderly in rural China are generally low. ***Second,*** in all SF-12 items, the HRQOL is low. ***Third,*** men’s scores are higher than that of women’s. The elderly with higher education reported higher scores than those with lower education. ***Fourth,*** the effect of socio-demographic variables of the rural Chinese empty-nest elderly on SF-12 scores is more significant, whereas the effect on EQ-5D scores is less significant.

## Introduction

With rapid economic development, extended average life expectancy, and further reduced fertility, China stepped into an aging society at the end of the 20th century. Statistical results of the Sixth National Census data in 2010 show that the population of people aged 65 and over accounted for 8.87% of the total, increasing by 1.91% of the Fifth National Census data in 2000 [1]. Such data describe a gradually accelerated aging process in China. Accompanied with the rapid development of aging in China, many older people have also been under a situation called "empty nest". Empty-nest older people are those who do not live with their children or do not have children. The reasons leading to the situation are diverse. One reason is the willingness of the elderly to choose a freer life. Another reason is the possibility that his/her children and/or relatives abandoned him/her, or the elderly was left alone at home because his/her children left for another city to earn a living [2]. The phenomenon of empty nest has become a rapidly rising trend among the elderly in the past decade and has become an important social issue that cannot be ignored in the aging process in China.

**Figure 1:**
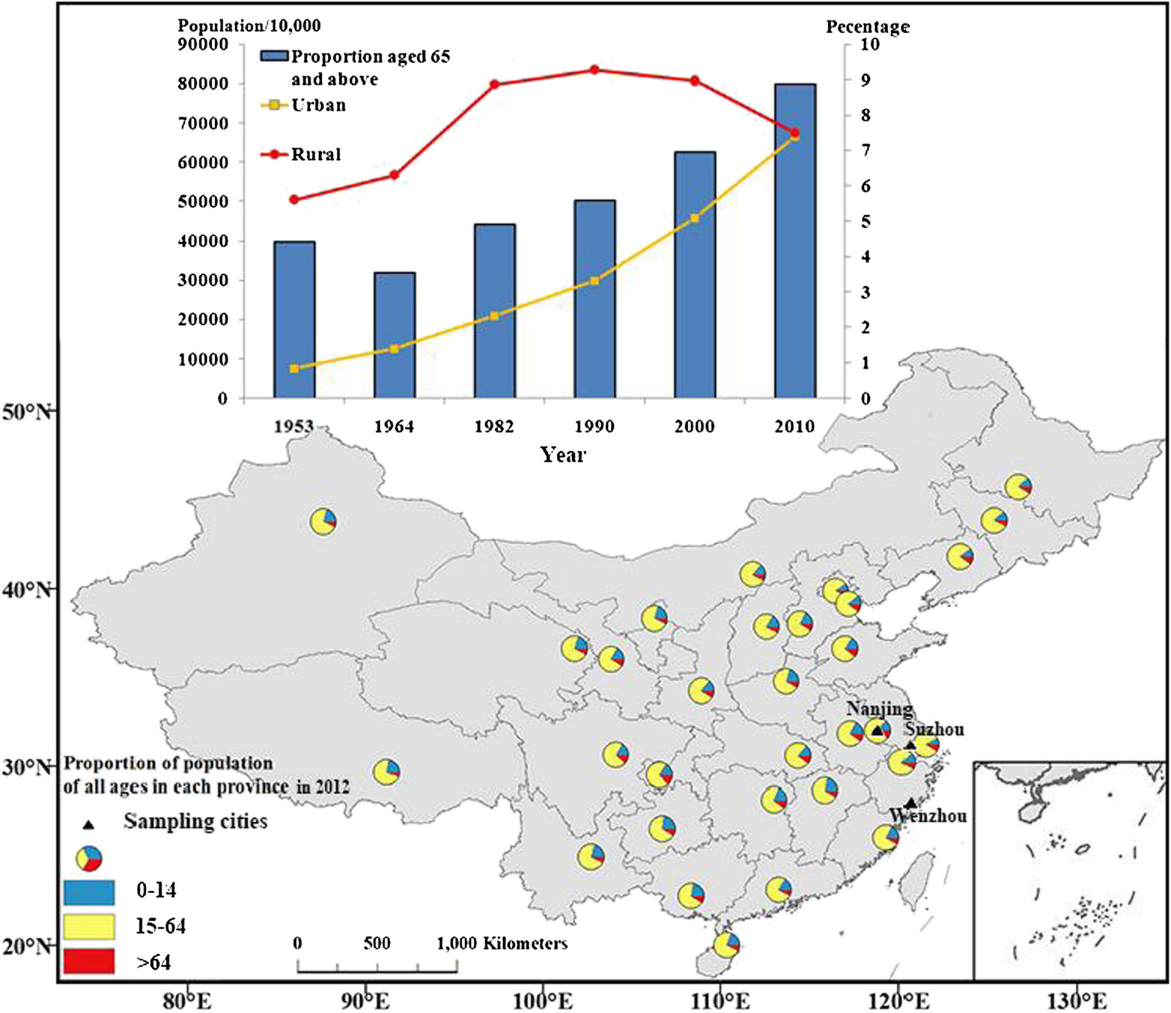
Changes in the population since the P. R. China was founded (Data resources: National Bureau of Statistics of the People’s Republic of China).

Figure 1 shows the change in population since the founding of the People’s Republic of China. The urban population started to grow rapidly since the start of the revolution and the implementation of the open-policy in the 1980s. By contrast, the number of the rural population decreased. By the sixth national census in 2010, the populations of the rural and urban areas were nearly the same (rural population = 674.15 million, urban population = 665.58 million). Meanwhile, the percentage of the elderly (aged 65 and above) reached 8.87% in 2010. The small pie charts in the map of China indicate the proportion of population of all ages in each province in 2012. The national sampling investigation data in 2012 showed that the percentage of the elderly in many provinces rose to 10%. The young adults in the rural areas went to work or lived in the cities, which resulted in an increase in the urban population, whereas fewer people stayed in the rural areas. The number of elderlies increased, but the number of people left to take care of the elderly decreased. This trend resulted in the growing number of empty-nest elderly in Chinese society.

The social pension system in China is not perfect, especially in the rural areas. Raising the elderly in a family remains the norm in rural areas, but more and more empty-nest elderly are facing uncertainty in terms of financial support, life care, and emotional comfort [3]. The elderlies who have physical disabilities, who live alone and do not receive care may cause health problems. Therefore, the health conditions of the rural Chinese empty-nest elderly should be addressed.

### Health of the elderly in rural China

Health issues of older people in rural China have caught the concern and interests of researchers^1^. Currently, research results mainly focus on three aspects.

***First***, Depression or loneliness is common among older people in rural China. 78.1% of the elderly in rural Anhui had moderate to severe levels of loneliness [4]. 26.5% and 4.3% of older people in rural Shandong and Sichuan met the criteria for mild and severe depression, respectively [5]. Risk factors related to loneliness or depression among older people in rural China are family function and social support [4], living alone and low cognitive function [5], poor self-rated health and presence of chronic disease [6], financial status, living arrangements, and some demographic factors [7].

***Second***, Mental health is poor among older people in rural China. Older parents with more migrant children tended to have significantly more depression and lower life satisfaction [8]. Older adults consider family support agreement as necessary, thus rendering the elderly more in need of parental support but less likely to get it [9].

***Third***, The quality of life (QOL) of older people in rural China is worrisome. One report use the 36-item Short Form Health Survey (SF-36) to evaluate the QOL in the older Chinese in Zhejiang, and all scale scores of SF-36 in the rural population were significantly lower than those in the urban population, except for the general health domain [10]. The Chinese elderly in rural Anhui were found that 49.7% of them slept poorly; poor physical component summary (PCS) and poor mental component summary (MCS) are two predictors for poor sleep quality [11]. Another research further emphasized this worrisome situation: the over-65 age populations report the highest rate of completed suicide, reaching 44.3 to 200 per 100,000, which is four to five times higher than the general population, and the rural suicide rate is three to five times higher than the urban suicide rate [12].

The above literatures focused on the large population of the rural Chinese elderly. Existing literature suggests that the health of the rural Chinese elderly, especially the empty-nest elderly who are a more vulnerable group, is a cause for worry. The empty-nest elderly encounters problems in material deprivation and psychological issues. The empty-nest elderly will encounter difficulty in addressing this reality on their own. The problem of rural Chinese empty-nest elderly is not only a specific problem of individual family, but is also an extensive issue in the society. Thus, research is needed on the health of the rural Chinese empty-nest elderly.

### Health condition of empty-nest elderly Chinese

As a more vulnerable group in China, the empty-nest elderly have been given great concern. The current studies are mainly focused on three aspects.

***First***, The living arrangements of the empty-nest elderly are worrisome. A study showed that majority of the Chinese elderly lived independently or in "empty nests,". Housing and community facilities for the elderly were also insufficient (particularly those serving the disabled) [13].

***Second***, Many empty-nest elderly suffer from loneliness or depression. A survey in a mountainous county found that the empty-nest elderly had lower life satisfaction, less social support, higher prevalence of chronic diseases, more feelings of depression and loneliness, and lower physical and mental health scores than not-empty-nest elderly. The empty-nest elderly were also likely to have mental health problems and dissatisfaction with life [14], [15]. The determined prevalence of depression of the empty-nest elderly was 74.46%, indicating a higher level [16]. Furthermore, if the empty-nest elderly were better supported and looked after, their negative mental issues might be prevented or at least relieved [17]. A geriatric depression scale was applied to investigate the empty-nest elderly in Hunan and found that the average score of the rural group is higher than that of the urban group, indicating a worse situation [18]. 30.11% of the empty-nest elderly in rural Sichuan showed anxiety-related symptoms of anxiety disorders and that depression [19].

***Third***, The health of the empty-nest elderly is deteriorating. One study on the Chinese elderly showed that living alone is associated with low subjective well-being, and living with immediate family is related with positive subjective well-being [20]. The empty-nest elderly in a mountainous area were less likely to call for doctor because they lack financial and social support [21]. And the empty-nest elderly in Shanghai were more likely to have lower social support and worse self-reported health than the non-empty-nest group [22]. Another study showed that the empty-nest elderly in Shanghai had less social support and higher prevalence of chronic diseases and had higher home care needs than the non-empty-nest elderly [23]. Some scholars used the SF-36 on the empty-nest elderly and found that scores of the scale and the psychological domain were significantly low [24].

The negative health of the empty-nest elderly should arouse our vigilance and strong attention. However, existing literature focuses on specific psychological health and living conditions with extensive meaning and does not explore the general aspects of health. In studying the issue of the empty-nest elderly, Chinese scholars are more concerned on the urban elderly than the rural ones [25]. This highlights the urgency to study the rural Chinese empty-nest elderly.

### Health-related quality of life (HRQOL) and measuring scales

The HRQOL is related to both self-reported chronic diseases (e.g., diabetes, breast cancer, arthritics, and hypertension) and risk factors (e.g., body mass index, physical activities, and smoking status). Measuring the HRQOL can help determine the burden of preventable diseases, injuries, and disabilities. This measurement can help provide valuable new insights into the relationships between HRQOL and risk factors. Measuring the HRQOL can also help monitor progress in achieving the nation’s health objectives [26]. HRQOL can test the effects of various health changes and provide further references for clinical management and policy decisions [27]. Many scales can be used to measure the HRQOL [28]. Excellent standard lies in obtaining semantic, idiomatic, experiential, and conceptual equivalence in translation, pre-testing techniques, and re-examination of scores [29]. The scales used to measure Chinese HRQOL are SF-36 [30,31], World Health Organization Quality of Life BREF (WHOQOL-BREF) [32,33], and others. Most of these measures have good applicability. This study uses the five-domainal European quality of health scale (EQ-5D) and the12-item Short Form Health Survey (SF-12).

EQ-5D is a handy, easy-to-use tool for measuring health outcomes that has been increasingly used in health services, economic analyses, pharmacies, and population health surveys. It is widely used to assess and test the quality and effectiveness of health services. The validity and reliability of EQ-5D have been tested in populations in mainland China [34-37]. It has also been applied to many populations to measure their HRQOL [38], [39].

SF-12 is a subset of SF-36. They both help evaluate the quality of care in managed care plans and other health care applications. They have also been widely used and extensively validated in clinical settings and special populations [40]. Moreover, tests of reliability and validity have been taken, and the results show the practical significance of the SF-12 scale. SF-12 can be a practical alternative to the SF-36 for large group comparisons with a focus on overall physical and mental health outcomes [41-43]. The SF-12 scale is popular as it improves efficiency and lowers the costs for profiles and summary scales. This study also uses the SF-12 scale as the samples in this study are large enough. Researches have indicated that Chinese version of SF-12 is an effective measurement tool to Chinese population [44]. It is feasible and credible [45].

Strong comparability exists between SF-12 and EQ-5D scales in measuring the health burden of chronic diseases, recent health problems, and social inequity [46]. Both measures can retain consistency when measuring the sociological demographic differences of population health [47]. However, EQ-5D has an important ceiling effect. A combination with SF-12 can provide more range in measuring health [48]. Considering that SF-12 and EQ-5D are internationally acknowledged mature scales and their combination produces an advantage, this study will simultaneously use SF-12 and EQ-5D to measure the HRQOL of the rural Chinese elderly.

### Research objectives

This study takes the cities of Nanjing, Suzhou, and Wenzhou as examples. We use the EQ-5D and the SF-12 to measure the HRQOL of empty-nest elderly living in the rural areas of the three cities, which represent the economically developed areas in China. Research results may not only provide practical significance to the challenges of rural aging population but also further reference for other developed areas in China. Studying the HRQOL of empty-nest rural elderly and improving their HRQOL are of great practical significance as the HRQOL is an important part of building new socialist villages and is related to the creation of a harmonious society in rural China.

This study aims to (1) provide the basic information of the rural Chinese empty-nest elderly and detailed evidence of different domains of HRQOL as reference for future studies; (2) explore the social reasons that lead to the HRQOL situation of the rural Chinese empty-nest elderly to facilitate policy making for governmental and social organizations on how to improve the HRQOL; (3) compare the differences of the HRQOL measured by different scales and provide practical evidences of the application of SF-12 and EQ-5D on the rural Chinese empty-nest elderly population.

## Methods

### Sampling and respondents

The study explores the HRQOL of the empty-nest elderly in rural China. A questionnaire investigation is conducted on the empty-nest rural elderly aged 60 and over in three cities: Nanjing, Suzhou, and Wenzhou. Creating a complete sampling frame without any mission is impossible because of the large jurisdiction areas of three cities with large numbers of empty-nest elderly. Therefore, we used the multi-stage sampling method. The sampling units used in China at the early stage are mainly the administrative units or organizations [49]. First, two counties/cities are randomly selected from each city. Gaochun County and Lishui County were chosen from Nanjing; Wujiang City and Taicang City were chosen from Suzhou; Pingyang County and Cangnan County were chosen from Wenzhou. Second, two towns are randomly selected from each county/city. Third, three villages are randomly selected from each town.

Finally, the amended sampling with Probability Proportionate to Size (PPS) is used to compensate for the errors or defects that may occur in the multi-stage sampling process [49]. The PPS sampling method ensures that the probability is proportional to the size of elements. This study used the PPS method to determine the sample size of each village according to the demographic data of the Villagers’ Committees or the local Civil Affairs Bureau because the distribution of the empty-nest elderly is not the same in different villages. The Villagers’ Committees or the local Civil Affairs Bureau provides the basic information of the empty-nest elderly, including their names and addresses. The investigators randomly chose the household that met the investigation requirement. The investigators visited their homes to ask for permission. The investigation will be conducted with their consent. If they refuse, the investigator will go to another family until enough respondents were found to satisfy the sample size determined by PPS method. The respondents verbally expressed their willingness to participate in the investigation.

All questionnaires were completed by the investigators according to the answers of the respondents through one-to-one live interview. Those who were unconscious or had slurred speech were excluded from the investigation. All investigators had to undergo uniform training prior to the investigation. During the investigation, the questionnaire used a unified guidance language and unified description of the ambiguous items. The questionnaires were completed anonymously and received on the spot. The investigation was conducted in the summer of 2013. A total of 1200 questionnaires were distributed and 967 valid ones were received. The effective rate was 80.58%.

The questionnaires include basic information of the empty-nest elderly: gender, age, and education. A total of 424 men participated in the survey, accounting for 44.3% of the total number, and 534 women participated, accounting for 55.7% of the total number. The minimum age of the respondents was 60 and the maximum age was 98. The mean value and standard deviation of the ages were 78.27 and 9.585, respectively. On the basis of the skewness and kurtosis values (kurtosis = -1.024; skewness = -0.162), the age distribution was negatively biased and not as steep as the standard normal distribution. In terms of education distribution, the illiteracy percentage of older empty-nest Chinese elderly was the highest, accounting for 39% of the total. Primary school ranked second at 36.4%. The Respondents with a high school education or above only accounted for 9.6% of the total.

### Instruments

#### EQ-5D

The EQ-5D has two components: the EQ-5D health state description system and the EQ-visual analogue scale (EQ-VAS) scores. The EQ-5D health state description system has five domains: mobility (M), self-care ability (SC), usual activities (UA), pain/discomfort (PD), and anxiety/depression (AD). Each domain has three choices: none, moderate, and severe/extreme (coded 1, 2, and 3, respectively). For example, "11111" indicates that the respondents have no health problems in all five domains (i.e., the best health condition), and "33333" indicates that the respondent have severe health problems in all five domains (i.e., the worst health condition). In terms of "unconscious" and "death" in both cases, the EQ-5D has 245 kinds of health conditions. EQ-VAS is a visual graduated scale, with the top 100 points representing the best health in mind and the bottom of 0 points representing the worst health in mind.

#### SF-12

The SF-12 scale is a simplified version of the SF-36 with 12 items and eight domains, namely, physical function (PF), physical role (RP), bodily pain (BP), general health (GH), vitality (VT), social function (SF), emotional role (RE), and mental health (MH). Using a standard scoring method, we can calculate the general score of PCS and MCS. PCS includes PF, RP, BP and GH; MCS includes RE, SF, MH and VT.

#### Reliability analysis of scales (Cronbach’s)

Cronbach’s α coefficient is usually used when studying the reliability of scales. Generally speaking, a reliability coefficient above 0.9 indicates good reliability. Reliability above 0.8 is considered acceptable, whereas above 0.7 indicates that the questionnaire is valuable but should be revised, and below 0.7 indicates low reliability. Table 1 shows the reliability analysis of the SF-12 and EQ-5D scales. The Cronbach’s α of EQ-5D is 0.775 (>0.7), and that of PCS and MCS are 0.713 (>0.7) and 0.763 (>0.7), respectively. These results indicate that the scales have certain reference values and worthy of further research. Table 1 shows the Cronbach’s α in all domains.

**Table 1:** **Cronbach’s** α **coefficients**

**Items**		**Reliability coefficient**	
		**Domains**	**Cronbach’s α**
EQ-5D		M	0.721
		SC	0.736
		UA	0.740
		PD	0.737
		AD	0.737
SF-12	PCS	PF	0.627
		RP	0.636
		BP	0.645
		GH	0.703
	MCS	VT	0752
		RE	0.652
		SF	0.739
		MH	0.665

Notes: the outcome is the α of EQ-5D and SF-12. In SF-12, PF refers to physical functioning; RP refers to role-physical; BP refers to bodily pain; GH refers to general health; VT refers to vitality; SF refers to social functioning; RE refers to role-emotional; MH refers to mental health. In EQ-5D, M refers to mobility; SC refers to self-care; UA refers to usual activities; PD refers to pain/discomfort; AD refers to anxiety/depression.

### Research methods

#### Scoring methods of EQ-5D

The earliest British EQ-5D utility scoring system provides an important model for the study of other countries. Its results are recommended for other countries or regions that do not have his system. However, considering the realistic differences in the national social and cultural backgrounds, we used the Japanese scale utility scoring system: U = 1-(0.152 + 0.075*M2 + 0.418*M3 + 0.054*S2 + 0.102*S3 + 0.044*U2 + 0.133*U3 + 0.080*P2 + 0.194*P3 + 0.063*A2 + 0.012*A3). M2 to A3 are the 10 main variables. M2, S2, U2, P2, and A2 represent the mobility, self-care ability, usual activities, pain/discomfort, and anxiety/depression, respectively, in the second level (code 1); otherwise the code is 0. M3, S3, U3, P3, and A3 represent the mobility, self-care ability, usual activities, pain/discomfort, and anxiety/depression in the third level (code 1); otherwise the code is 0.

#### Scoring methods of SF-12

This study used a standard scoring method. Details can be referred to a professional book [50]. In scoring the eight domains and two domains in the SF-12 in this study, the total scores in each domain were transferred to the hundred mark system, and the Z-scores of the eight domains and two domains were calculated. The formula Score = 50+ (domain/Z-value (domain) × 10) was used to calculate the standard score.

#### Analysis methods

The reliability analysis is conducted on the scales first. The scores of the scales will then be changed according to the formula. The basic information of the scales will be analyzed through the bar chart and line chart. Finally, the Spearman correlation coefficient, stereotype logistic regression, ordered probit regression, multinomial logistic regression, and SEM methods are employed to study the correlations between the independent variables (e.g., age, gender, education, marriage) and scales.

The stereotype logistic regression is the improvement of traditional ordered logit models. This model does not retain the information of the rank order of the dependent variables, but it also does not need the assumption that the curve slopes of categorial regression are the same. This model is flexible and practical, which is rendered in the following formula:

$$\text{Pr}\left(Y=y_{s}|x\right)=\frac{\exp\left(\alpha_{s}+\Phi_{s}\beta^{\prime }x\right)}{\sum_{l=1}^{k}\exp\left(\alpha_{l}+\Phi_{l}\beta^{\prime }x\right)},\;s=1,\ldots ,k$$

The coefficient to be estimated of independent variable x is denoted by β. The intercept point of the dependent variable is denoted by "k," α_s_, α_l_ are the intercept of the model. The sequenced regression relationships can be obtained by defining a monotonically increasing Φ. If the coefficient of the model is positive, the characteristic has a positive impact on the dependent variable. By contrast, if the coefficient of the model is negative, the characteristic has negative impact on improving the HRQOL of the empty-nest elderly.

The multinomial logistic model does not require that the parallel regression assumption or the relationships of rank orders of the dependent variables should meet. The correlations between EQ-5D and the independent variables can be analyzed. In multinomial logistic model, logits are formed from contrasts of category pairs of the dependent variable. Each logit is then modeled in a separate equation.

Structural equation model (SEM) shows the relationship between latent variables and measurable variables through figures. These variables can handle several dependent variables simultaneously, and allow independent variables and dependent variables to commit measurement errors. These variables also estimate the relationship between factors and factors structure, and allow the measurement of models with more flexibility.

These variables cab present the research idea more clearly and concisely and give effective supplements to the regression results. The added SEM not only contains the relationships between demographic control variables and scales but also further research the coefficients of all domains.

## Results

### Descriptive analysis of EQ-5D

**Figure 2:**
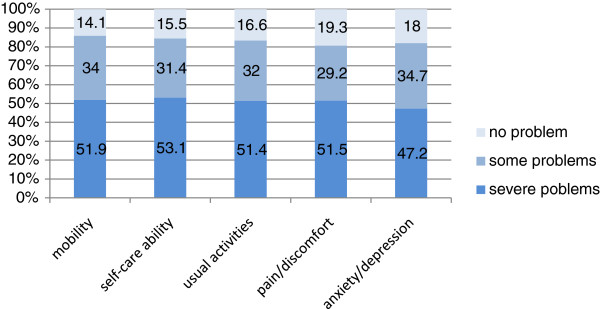
Distribution across the severity levels of the EQ-5D domains at baseline.

Figure 2 shows the EQ-5D scores of the HRQOL of the empty-nest Chinese elderly. The respondents with "severe problems" accounted for the highest percentage, followed by those with "some problems." Only a few respondents chose "no problem." In the domain of mobility, 51.9% of the elderly could not ambulate, and 34% had inconvenience in some actions. Only 14.1% of the elderly choose "I have no problems in walking about;" this percentage is the smallest compared with those of the other four same-level items. In the domain of self-care ability, 53.1% of the respondents could not wash faces, take a bath, and dress themselves up, accounting for the greatest proportion among all same-level items. About 31.4% of the elderly had problems in self-care ability, and 15.5% had no problems.

In the domain of usual activities, 51.4% of the respondents could not conduct any usual activities, 32% had some problems, and 16.6% had no problems. In the pain/discomfort domain, 51.5% had severe pain/discomfort, 29.2% had some pain/discomfort, which is the lowest percentage among all same-level items, and 19.3% had no pain/discomfort, which is the highest percentage among all same-level items. Anxiety/depression reflects the mental condition of the empty-nest Chinese elderly. Among the respondents, 47.2% had severe anxiety/depression, which is the lowest percentage among all same-level items, 34.7% had some problems, and 18% had no symptoms of anxiety/depression. Overall, the HRQOL of the respondents was relatively low and the gap between the five domains was small.

**Figure 3:**
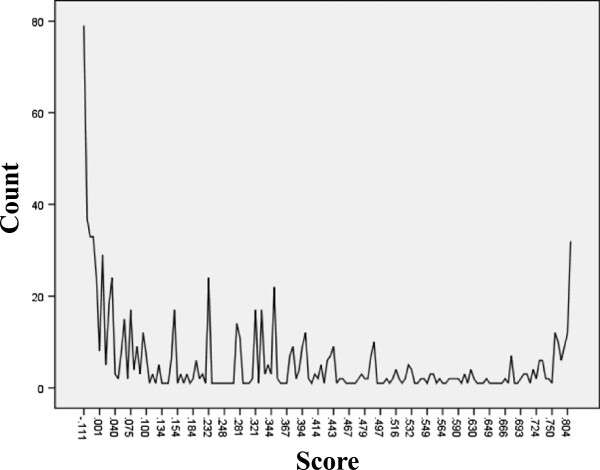
Utility score curve.

Figure 3 shows the utility score curve based on the calculation of the EQ-5D sores of the empty-nest Chinese elderly using the Japanese scale utility systems. With a score of 0.848, "11111" denotes that the health condition of the elderly is the best. With a score of -0.111, "33333" indicates that the health condition of the elderly is the worst. As illustrated in Figure 3, the curve is U-shaped distributed, indicating that the health of the elderly shows polarization. Both of the best and the worst conditions account for the majority, and the figure indicating the respondents who considered themselves to have the worst health is large. Overall, the effective score curve declines with the scores. This result can be interpreted as the percentage of worst health condition being higher than that of the best health condition. This phenomenon is also obvious in the 0.47 score. Moreover, the utility score curve is steep in the score section of -0.111 to 0.47 and gentle in the scores section 0.47 to 0.848. Therefore, the gap in the respondents with the worst health condition is relatively large but has differences in the five domains. The gap in the respondents with the best health condition is relatively small and is positive in all five domains.

### Descriptive analysis of SF-12

**Figure 4:**
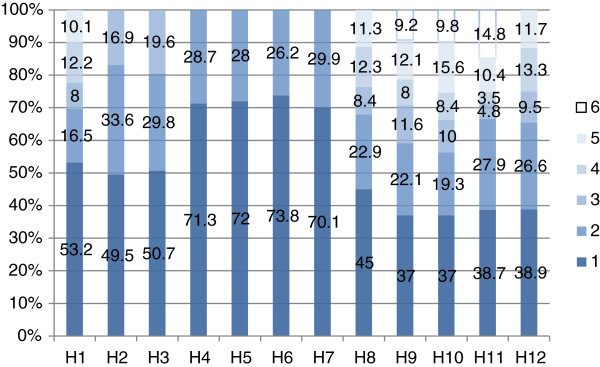
Distribution across severity levels of the SF-12 domains at baseline.

Figure 4 shows the SF-12 of the HRQOL of the respondents. H1 indicates "how do you feel your current health condition." The lightest color denotes very good health, and the darkest color indicates very poor state of health. As shown in the first column of Figure 4, 53.2% of the respondents reported very poor health condition, and only 10.1% were very satisfied with their health. H2 indicates "does your health limit the activities." The lightest color indicates no limit, and the darkest color denotes a large limit. As shown in the second column, 49.5% of the respondents could not do appropriate activities because of their physical health, and 16.9% had no limitation. H3 indicates "how many flights of stairs can you climb," H4 indicates "if you accomplish less work than you would like," H5 indicates "are limited in any kind of work or other activities," H6 indicates "cut down the amount of time you spend on work or other activities," H7 indicates "do not work or perform other regular daily activities as carefully as before," H8 indicates "have problems with your work or other activities as a result of your physical health in the past four weeks," H9 indicates "are calm and peaceful," H10 indicates "do you have a lot of energy," H11 indicates "are down hearted and depressed," and H12 indicates "how much has your physical health or emotional problem interfered with your social activities (e.g., visiting friends, relatives, etc.)." As shown in Figure 4, the poor situation accounts for a large proportion in all 12 items, and the good condition accounts for a small one. Thus, the HRQOL scores of the empty-nest Chinese elderly through the analysis of SF-12 scale are low.

### EQ-5D and SF-12

#### Multidimensional Spearman correlation coefficient analysis

Table 2 shows the analysis of the multidimensional Spearman correlation coefficient. In general, all domains show slight correlation and all pass the significance test. A relatively strong correlation, such as the PF and MH (Spearman coefficient = 0.438), RE and AD (Spearman coefficient = 0.447), and RE and PD (Spearman coefficient = 0.443) is observed in similar domains. VT domain only exists in SF-12 and has no direct relations with any items in EQ-5D. Therefore, the correlations with EQ-5D show a series of low correlation coefficients. For example, the correlation coefficients between VT and UA, PD, and AD are below 0.300.

**Table 2:** Multidimensional Spearman correlation coefficient analysis of all domains

		**SF-12 domains**							
		**PF**	**RP**	**BP**	**GH**	**VT**	**RE**	**SF**	**MH**
EQ-5D domains	M	0.438**	0.401**	0.331**	0.330**	0.318**	0.440**	0.306**	0.432**
	SC	0.386**	0.397**	0.335**	0.331**	0.314**	0.442**	0.414**	0.415**
	UA	0.418**	0.422**	0.367**	0.312**	0.290**	0.425**	0.341**	0.385**
	PD	0.404**	0.372**	0.356**	0.305**	0.267**	0.443**	0.293**	0.423**
	AD	0.396**	0.344**	0.322**	0.304**	0.260**	0.447**	0.331**	0.335**

Note:** indicates that the correlations are significant when degree of confidence (bilateral) is 0.01. In SF-12, PF refers to physical functioning; RP refers to role-physical; BP refers to bodily pain; GH refers to general health; VT refers to vitality; SF refers to social functioning; RE refers to role-emotional; MH refers to mental health. In EQ-5D, M refers to mobility; SC refers to self-care; UA refers to usual activities; PD refers to pain/discomfort; AD refers to anxiety/depression.

#### Regression

##### Analysis methods of Model 1–5

Table 3 shows five models. The dependent variables are PCS, PF, RP, BP, and GH, respectively. Before choosing the regression models, the dependent variables are classified according to the method of the mean plus or minus standard deviation. The low-level HRQOL is coded as 1, the middle-level HRQOL is coded as 2, and the high-level HRQOL is coded as 3. Thus, the higher the level is, the higher the HRQOL score of the rural Chinese empty-nest elderly is. PCS and PF can be divided into three categories. The stereotype logistic model is used to conduct the regression analysis. Other variables can only be divided into two categories (middle-level and high-level) because of the sampling errors. Thus, the ordered probit models are used to conduct the analysis.

**Table 3:** Ordered probit regression and stereotype logistic regression of PCS and related domains

	**Model 1**	**Model 2**	**Model 3**	**Model 4**	**Model 5**
	**PCS**	**PF**	**RP**	**BP**	**GH**
Gender (Female = 0)	0.200	0.124	-0.010	0.054	0.254*
Age	-0.020*	-0.054*	-0.023	0.000	-0.000
Primary school (illiteracy = 0)	0.035	1.750***	0.900*	0.040	-0.214
Middle school (illiteracy = 0)	0.008	4.127***	2.011***	1.318***	0.735***
High school or junior colleges and above (illiteracy = 0)	4.105	6.922***	2.845***	2.303***	0.984***
Not widowed (others = 0)	-0.615*	-0.900	0.111	-0.311	0.025
Widowed (others = 0))	-0.601*	-0.711	0.038	-0.470*	-0.154
Df	7	7	7	7	7
Wald Chi (2)		193.86			
LR Chi (2)	43.65		207.65	279.88	127.88

Note: ***indicates p < 0. 001; **p < 0.01; *p < 0.05. PF refers to physical functioning; RP refers to role-physical; BP refers to bodily pain; GH refers to general health.

(1) PCS model

The age and marital status variables pass the significance test in model 1. The age variable has a negative impact on the PCS scores, which indicates that the PCS scores decline with age. The result is consistent with the general understanding. The organ function and the health status of the elderly deteriorate with age. Therefore, the scores of physical health decline. The odd ratio (OR) results of education and marital status show that the probability of PCS scores improve one level for elderly who were not widowed, which is 0.541 times higher than that of elderly who were divorced. The probability of PCS scores of widowed elderly to improve one level is 0.548 higher than the elderly who were divorced. The results indicate that the PCS scores of widowed elderly or divorced elderly are higher.

(2) Domains of PCS

The education variable in models 2, 3, 4, and 5 does not pass the significance test. Results show that the higher the education of the elderly, the higher are their scores in PH, RP, BP, and GH. The four domains are indicators to measure the physical health of the empty-nest elderly. Higher score indicates better physical health. This result may be attributed to the higher possibility of elderly with higher education to engage in less intensive physical work and engage in more physical exercise.

The gender variable in model 5 passes the significance test with an OR of 1.289. The probability of the men’s GH to improve one level is 1.289 higher than that of women’s, which indicates that men’s GH scores are higher than that of women’s.

##### Analysis methods of model 6 to 10

Table 4 provides five models. The independent variables are MCS, VT, RE, SF, and MH, respectively. MCS, VT and MH can be divided into three categories by method of the mean value plus or minus standard deviation. The stereotype logistic model is used to conduct the regression analysis. Other dependent variables can only be divided into two categories (middle-level and high-level) because of sampling errors, thus the ordered probit model is employed.

**Table 4:** Ordered probit and stereotype logistic regression on MCS and related domains

	**Model 6**	**Model 7**	**Model 8**	**Model 9**	**Model 10**
	**MCS**	**VT**	**RE**	**SF**	**MH**
Gender (Female = 0)	0.0687	0.118	0.031	0.026	-0.105
Age	-0.007	0.003	0.028	-0.008	0.052*
Primary school (illiteracy = 0)	1.630	0.552	3.866	0.228*	1.461***
Middle school (illiteracy = 0)	7.353***	2.939	7.017	1.347***	7.729***
High school or junior colleges and above (illiteracy = 0)	8.713***	4.056	7.573	1.845***	10.192***
Not widowed (others = 0)	-0.071	-0.246	-0.536	-0.080	-0.376
Widowed (others = 0))	0.465	-0.174	-0.095	-0.032	-0.112
Df	7	7	7	7	7
Wald Chi (2)	98.36	120.16			94.90
LR Chi (2)			538.77	309.37	

Note: ***indicates p < 0.001; **p <0.01; *p <0.05. VT refers to vitality; SF refers to social functioning; RE refers to role-emotional; MH refers to mental health.

(1) MCS model

The education variable passes the significance test in model 6. Results indicate that the scores of the illiterate elderly are lower than those elderlies who went to middle school, high school, junior college, or higher. The higher the education level is, the higher the MCS scores are. Elderly with higher education may have a relatively rich cultural life, which has a positive impact on psychological health.

##### Domains of MCS

No independent variables pass the significance test in models 7 and 8. The education variable passes the significance test in model 9. Results indicate that the elderly with higher education performs more social roles, and the social values they produce are higher than those with lower education, therefore their SF scores are higher.

Apart from the education variable, the age variable also passes the significance test in model 10. Results show that the older the elderly is, the higher their MH score is. With the age, the elderly has more experiences in dealing with the interpersonal relationships and social affairs. Their psychological health becomes calmer. Thus, they have higher MCS scores than the younger elderly.

#### Analysis methods of EQ-5D

The EQ-5D scale contains five questions. Each question consists of three options. The first option can be generalized as "no problem;" the second option can be generalized as "some problems;" and the third option can be generalized as "extreme problems." Multinomial models are employed to conduct the regression analysis because the hierarchy between options is not obvious. Regression results are shown in Table 5. The last category in five models is considered as the reference category.

(1) M model

**Table 5:** Multinomial logistic regression of EQ-5D

		**M**	**SC**	**UA**	**PD**	**AD**
		**Coefficient**	**Coefficient**	**Coefficient**	**Coefficient**	**Coefficient**
No problem	Gender (Female = 0)	0.111	0.400	0.565	-0.015	0.553*
	Age	-0.017	-0.039	-0.055*	-0.039	0.002
	Primary school (illiteracy = 0)	1.152*	0.698	1.235**	1.188*	0.841*
	Middle school (illiteracy = 0)	3.537***	3.050***	3.197***	3.220***	3.458***
	High school or junior colleges and above (illiteracy = 0)	7.289***	6.072***	5.596***	5.876***	6.637***
	Not widowed (others = 0)	-0.491	0.086	0.296	0.133	0.002
	Widowed (others = 0))	-0.824	0.513	0.442	-0.146	0.421
Some problems	Gender (Female = 0)	-0.175	-0.015	0.012	-0.135	0.328
	Age	0.008	-0.006	0.008	-0.008	0.010
	Primary school (illiteracy = 0)	0.884***	0.321	0.962***	0.692***	0.742***
	Middle school (illiteracy = 0)	2.398***	1.593***	2.406***	1.812***	1.500***
	High school or junior colleges and above (illiteracy = 0)	4.479***	3.104***	3.832***	3.101***	4.218***
	Not widowed (others = 0)	0.003	-0.273	-0.003	0.008	0.355
	Widowed (others = 0))	0.014	-0.064	0.113	0.061	0.581

Note: ***indicates p < 0. 001; **p < 0.01; *p < 0. 05. In EQ-5D, M refers to mobility; SC refers to self-care; UA refers to usual activities; PD refers to pain/discomfort; AD refers to anxiety/depression.

The education variable passes the significance test when mobility and self-care ability are the dependent variables. The results are consistent with the SF-12, which indicates that elderly with higher education have fewer mobility problems.

(2) SC model

Elderly with higher education may perform less intensive physical work, which may have certain positive indications on physical health. Elderlies with good mobility are bound to have good self-care ability.

(3) UA model

The gender variable shows weak significance when "usual activities" is the dependent variable, compared with "no problem" with "extreme problems." Age and education variables pass the significance test. The Exp (B) of gender is 1.759, wherein the probability of men with "no problems" in usual activities is 1.759 higher than that of women’s. The Exp (B) of the age variable is 0.946, which indicates that difficulties in usual activities increase with age. The Exp (B) of the education variable increases as the gap between the comparison group and the reference group increases. This result indicates that the higher the education is, the fewer the difficulty there is in daily life. The results show a consistency in the results between PCS, PH, BP, RP, and GH.

(4) PD model

When pain/discomfort is the dependent variable, the education variable passes the significance test.

(5) AD model

When anxiety/depression is the dependent variable, the gender variable passes the significance test. The gender coefficient is 0.553 and the Exp (B) is 1.74, which indicate that the probability of the male elderly not feeling anxious/depressed is 1.74 times higher than that of the female elderly. The results illustrate that the psychological health of the female empty-nest elderly should be given more concern.

#### SEM analysis

**Figure 5:**
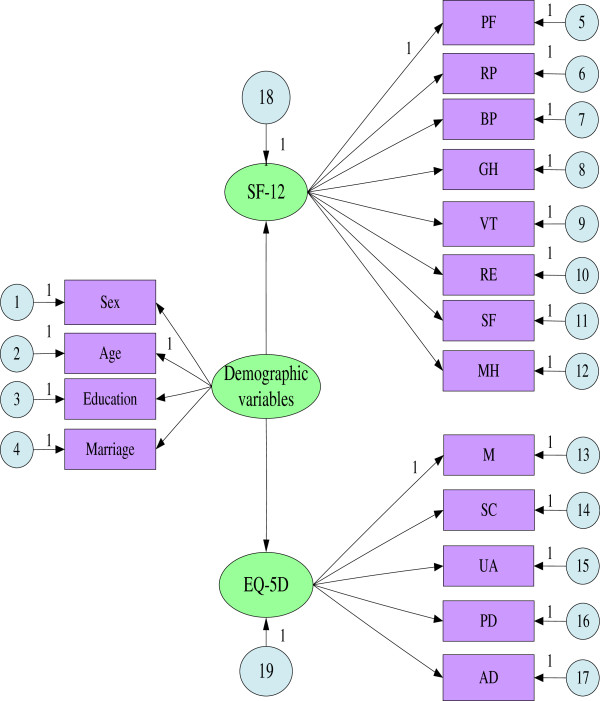
SEM analysis of SF-12 and EQ-5D.

Figure 5 shows the relationships between the four independent variables and SF-12 and EQ-5D. The model analysis results show that chi-square = 213.252; degrees of freedom (df) = 117; chi-square/df = 1.82 < 2, which indicates that the model fits the observed data. CFI = 0.986 and RMSEA = 0.029 indicate a good degree of a convergence measurement model and worthy of further research. Table 6 shows the results of the analysis.

**Table 6:** SEM analysis results

			**Estimate**	**SE**	**CR**	**P**
SF-12	<--	Demographic variables	-.962	.044	-21.832	***
EQ-5D	<--	Demographic variables	.064	.003	20.717	***
PF	<--	SF-12	1.000			
RP	<--	SF-12	1.213	.061	20.022	***
BP	<--	SF-12	1.199	.065	18.329	***
GH	<--	SF-12	1.057	.074	14.213	***
VT	<--	SF-12	.956	.063	15.261	***
RE	<--	SF-12	1.838	.080	23.089	***
SF	<--	SF-12	1.162	.065	17.929	***
MH	<--	SF-12	1.397	.064	21.825	***
M	<--	EQ-5D	1.000			
SC	<--	EQ-5D	.966	.054	17.930	***
UA	<--	EQ-5D	1.013	.055	18.286	***
PD	<--	EQ-5D	1.045	.057	18.209	***
AD	<--	EQ-5D	.929	.055	16.931	***
Marriage	<--	Demographic variables	.019	.003	6.872	***
Education	<--	Demographic variables	-.120	.004	-30.685	***
Age	<--	Demographic variables	1.000			
Sex	<--	Demographic variables	.036	.002	16.650	***

Note: ***indicates p < 0. 001; **p < 0.01; *p < 0. 05. In EQ-5D.

The results of Table 6 indicate that:

***First***, The correlation coefficient between SF-12 and the basic information is 0.962, and the correlation coefficient of EQ-5D is -.064. Both pass the significance test. The comparison suggests that the effect of socio-demographic variables (i.e., gender, age, education, marriage) of the rural Chinese empty-nest elderly on SF-12 scores is more significant, whereas the effect on EQ-5D scores is less significant.

***Second***, The correlation coefficients between SF-12 scores and eight domains show that the range of coefficients is 0.956 to 1.838. VT scores have the smallest impact on SF-12, whereas the RE scores have the greatest impact on SF-12. The correlation coefficient of RE is 1.838. That is, when RE increases each unit, SF-36 score increases 1.838 units.

***Third***, The correlation coefficients between EQ-5D scores and five domains show that the impact of each domain on EQ-5D is more balanced. These coefficients is maintained at about 1.000 and pass the significance test. For example, the correlation coefficient of SC is 0.966, whereas those of UA, and PD are 1.013 and 1.045, respectively.

***Fourth***, The relationship between the independent variables and demographic variables shows that both of them pass the significance test. Table 6 shows the gender coefficient is 0.036, which indicates that women’s scores are lower than that of men’s in demographic variables. The age coefficient is 1, which indicates that the scores of older elderly are lower than those of the younger elderly. The education coefficient is -0.120, which indicates that the scores of elderly with higher education are lower than those elderly with lower education. The marriage coefficient is 0.019, which indicates that widowed elderly or divorced elderly are lower than elderlies who were not widowed.

## Discussion

### Main findings

With the rapid development of China's economy for the past 30 years, the problems of the empty-nest rural elderly are becoming increasingly exposed. This study takes the rural areas in Nanjing, Suzhou and Wenzhou as samples and uses the EQ-5D and SF-12 scales to measure the HRQOL of the empty-nest rural elderly. It aims to determine the appropriate and effective measure to help improve the HRQOL of the empty-nest rural elderly and resolve the urgent population aging issue. The main findings of this study are as follows:

(1) On the basis of the answers to the EQ-5D scale, 50% of the respondents on average reported severe problems in all five domains and 30% had some problems. The EQ-5D scores show that the health condition of the respondents is polarized: respondents with the best and the worst health conditions account for the majority, and a large group of the respondents consider themselves to have the worst health. The gap in the respondents with the worst health conditions in the five domains is relatively large, and that in the respondents with the best health conditions is relatively small.

(2) On the basis of the answers to the SF-12 scale, the respondents generally obtained low scores. In all 12 items, the respondents who answered "poor" account for a large proportion, and those who answered "good" account for a small proportion.

(3) Finally, the comparison results through the Spearman correlation coefficient, regression, and SEM methods indicate that the correlations between the scales are high. The impact of the socio-demographic variables (i.e., gender, ages, education, marriage) on HRQOL varies with the dependent variables. The impact direction of gender and education on models are consistent. Male elderly report higher scores than women, and the elderly with higher education have higher scores.

### Reasons

Since the People’s Republic of China was founded in 1949, rural areas have experienced enormous changes. China has significantly accelerated its pace of urbanization. The urbanization rate increased from 17.9% in 1978 to 52.6% in 2012, with a sharp increase of 34.7% and an average annual increase of 1.0%. The total urban population has increased by 15,870,000 on average annually, and the total rural population has reduced by 4,350,000 on average annually. A large number of rural surplus labors have transferred to non-agricultural industries. In 2012, the number of rural-to-urban migrants in China reached 260,000,000 [51]. A large number of young people leave their villages and work in the city. Therefore, many elderly are left in the villages, resulting in the prominent phenomenon of empty nest.

With the development of economy, people’s living standard has dramatically improved in the past decades. This change is also particularly noticeable after the reform and opening up policy was implemented. Accordingly, the levels of health service and people’s health awareness have greatly improved. The life expectancy of residents increased from 67.8 in 1981 to 74.8 in 2010 [51]. However, their health conditions still deserve attention. The prevalence of mild cognitive impairment in the elderly in China was 9.6% and 14.7% in Eastern and Western China, respectively [52]. And the pooled prevalence of depressive symptoms in Chinese older adults is 23.6%, with single adults (i.e., divorced, unmarried, or widowed) having higher rate [53].

The growth of the Chinese elderly has brought many social problems, and its reasons and effects have attracted many discussions. Only a small percentage of healthy elderly did not work. Any true retirement is rare in rural China. In the lack of formal pension plans, older people must expend a great deal of effort to sustain the asset base of their own household and their children’s households. With the rapid increase in migration and increasing opportunities of households to move permanently to the city, older people have to work even more [54]. The one-child policy has a great impact on the growth of the elderly in China and is also producing profound many social and economic complications that require the development of appropriate policies [55-58]. On one hand, in rural China, the challenges in caring for children and the elderly are becoming more urgent, as the number of the elderly is increasing and the young have fewer choices. Many families are structured as 4-2-1 as the first generation of only children has reached their marrying age. A young married couple has to take care of two sets of parents without help from siblings [59]. On the other hand, as the regional economic development in China is not balanced, many young people choose to leave their villages and work or settle down in towns. Such rural-to-urban migration has greatly influenced the fertility rate [60]. Family or social support have important impact on the health of rural elderly [61]. The elderly in rural areas lack care, and this inattention to their well-being affects their physical and mental health [62].

### Policy implications

***First***, Upholding the traditional Chinese family arrangements (i.e., three generations in a family) may be the most effective solution. In other words, efforts should be made to prevent the elderly from suffering the empty-nest phenomenon [63]. Older parents living in three-generation households or with grandchildren in skipped-generation households have better psychological well-being. Receiving greater remittances from adult children and having stronger emotional cohesion with them. Both financial and emotional support improve their well-being of the elderly. Traditional family arrangements are beneficial in rural Chinese society because they indicate an achievement of a cultural ideal [64]. Some studies have also proved that rural grandparents who perceive their children as filial or their family as harmonious, those who receive instrumental support and less monetary support from their grandchildren are likely to have higher levels of life satisfaction or well-being than those who do not [65,66]. The loneliness felt by older adults is even reduced by the affection for and from their children, and perceptions of attachment to their children increase their psychological well-being [67].

***Second***, The empty-nest elderly must be given financial support. Providing basic social security investments are crucial to improve the psychological well-being and life satisfaction of the elderly [68]. The elderly Chinese with low social status tend to negatively rate their health [69]. The empty-nest phenomenon undermine family support and lack of money reduce the self-care abilities of adults, and inadequate services weaken social support [70].

The government has an important function in the process of policy implementation. The Rural Mutual Health Care (RMHC) was a social experiment that was conducted in one of China’s western provinces from 2003 to 2006, and it had a positive effect on the health status of the participants aged over 55 [71]. The New Rural Cooperative Medical Scheme (NRCMS) was established in 2003, and its coverage was high. By the end of 2012, a total of 2,566 counties in rural China carried out work on the NRCMS; the participation rate was 98.3% [51]. However, a study also showed that the coverage of NRCMS was high but not adequate to improve the access to in-patient care of the poor and the chronically ill [72]. Therefore, the current health schemes need to be improved. Moreover, the health of the elderly is not only affected by individual income but also by the income inequality [73]. China’s rural health crisis can be resolved through a fundamental rethinking of health provision across China, focusing specifically on the poorest, most remote parts of the nation [74]. Increased state investment in basic health provision should be more balanced.

***Third***, The capabilities of rural community care should be established [75], [76]. Having a community-based service delivery model is important. Social work practice will make China’s model more efficient and more sustainable [77]. We suggest that, ***first***, the government should provide the extremely poor rural elderly with some basic community-based services (e.g., people who would provide services such as doing housework, haircutting, maintaining property, providing assistance to see a doctor, and doing shopping) with no charge. ***Second***, the mutual potential of the rural community should be fully used. Neighbors and friends of the poor elderly are encouraged to help, and these people should be paid by the government. In this way, the poor elderly receive help from people they are familiar with, and they can save on expenses. ***Third***, professional workers, such as rural doctors, plumbers, and barbers, can be included in the service system to provide more professional services for the elderly. Moreover, the poor elderly should be provided free services by the government, and the empty-nest elderly with good economic situation can also choose to avail of extra services with low pay.

This study has some limitations. *First*, the empty-nest elderly living in Nanjing, Suzhou, and Wenzhou were chosen. These three sampling cities are relatively economically developed in China. Therefore, the results in this study only provide reference for other developed areas in China. For poor areas, particularly those in Western China, further research is expected. *Second*, this study only focused on the HRQOL of the empty-nest elderly in rural China. Future studies are suggested to explore the factors related to or are affected by the HRQOL. Moreover, more research on the empty-nest elderly living in urban areas is needed. *Third*, the empty-nest elderly is a large vulnerable group in China. If future studies could focus on long-term HRQOL, they may have an important role in the adjustment of national policies and government work.

## Conclusions

***First***, By analyzing the basic information, we conclude that the proportion of the respondents in rural areas is in accordance with the general rules. The age span of empty-nest rural elderly is large. The age distribution is negatively biased and not as steep as the standard normal distribution. The level of education of the empty-nest rural elderly is generally low, with illiteracy having the highest percentage.

***Second***, The EQ-5D shows the HRQOL of the empty-nest rural elderly. The respondents who answered "severe problems" account for a large proportion, and those who answered "no problem" account for a small proportion. Only a few respondents answered "some problems." The scores of the HRQOL of empty-nest rural elderly are relatively low.

The utility curve shows the polarization of the best and the worst conditions, which account for the majority. The number of respondents who reported very poor health condition is greater than those who reported very good health. The number of respondents who reported poor health is relatively large, and that of those who reported good health is small.

***Third***, SF-12 indicates the HRQOL of the empty-nest rural elderly. In all 12 items, the respondents who answered "poor" account for a large proportion, and those who answered "good" account for a small proportion. Therefore, the HRQOL of the empty-nest rural elderly is relatively low.

***Fourth***, The Spearman correlation coefficients indicate that the correlations of related domains between SF-12 and EQ-5D scales are strong. The correlations between different domains are relatively weak. All coefficients pass the significance tests.

Regression results show that men have higher scores of EQ-5D, PCS and MCS than women do. The older empty-nest elderly have lower scores of EQ-5D, PCS and MCS than the younger ones do. The elderly with higher education have higher scores of EQ-5D, PCS and MCS than those with lower education do. In addition, the gender and education differences of MCS scores are more obvious.

SEM analysis shows that Chi-square =213.252,df =117, Chi-square/df=1.82<2, indicating that the adaptation of observed model and practical data is very good. The basic information of empty-nest elderly has more significant impact on SF-12 scale.

## Competing interests

The authors declare that they have no competing interests.

## Authors’ contributions

YL wrote and revised the manuscript, was responsible for the design of the study, and performed the statistical analysis. WW participated in the statistical analysis. All authors read and approved the final manuscript.
